# Bayesian method for inferring the impact of geographical distance on intensity of communication

**DOI:** 10.1038/s41598-020-68583-1

**Published:** 2020-07-16

**Authors:** Fei Ozga, Jukka-Pekka Onnela, Victor DeGruttola

**Affiliations:** Department of Biostatistics, Harvard School of Public Health, Boston, MA 02115 USA

**Keywords:** Statistics, Statistical methods

## Abstract

Spatially-embedded networks represent a large class of real-world networks of great scientific and societal interest. For example, transportation networks (such as railways), communication networks (such as Internet routers), and biological networks (such as fungal foraging networks) are all spatially embedded. Both the density of interactions (presence of edges) and intensity of interactions (edge weights) are typically found to decrease as a function of spatial separation of nodes in these networks. Communication and mobility of groups of individuals have also been shown to decline with their spatial separation, and the so-called gravity model postulates that this decline takes the form of a power-law holding at all distances. There is however some evidence that the rate of decline might change as the distance increases beyond a certain value, called a change point, but there have been few statistically principled methods for determining the existence and location of change points or assessing the change in intensity of interactions associated with them. We introduce such a method within the Bayesian paradigm and apply it to anonymized mobile call detail records (CDRs). Our results are potentially useful in settings where understanding social and spatial mixing of people is important, such as in the design of cluster randomized trials for studying interventions for infectious diseases, but we also anticipate the method to be useful for investigating more generally how distance may affect tie strengths in general in spatially embedded networks.

## Introduction

Spatially embedded networks are networks in which each node has been assigned a fixed location in some underlying Euclidean space. Although this description could include embedding of nodes in a covariate space (e.g., representing fitness of nodes), here we focus on geographically embedded networks, i.e., networks that have been embedded in a two-dimensional Euclidean space where the positions of the nodes can be interpreted as geographical locations. Although this interpretation is not necessary for the formulation or use of the method, it applies to our specific application.

With the rise of communication and social network technologies, the role of spatial distance on establishing and maintaining social ties is constantly changing^1–3^. Knowing that two individuals communicate with one another using a specific channel or mode of communication makes them more likely to use also another^4–6^. For example, people who speak on the phone frequently also interact in person^7^. For researchers studying infectious diseases, such as HIV/AIDS or Malaria, the structure of social interactions in a population can provide valuable insights into how pathogens are transmitted among members of that population^8–10^. Another context for which the interplay between social ties and geography is important is in the delivery of healthcare. Patterns of care delivery can be naturally represented as networks, wherein two physicians are connected to one another if they share one or more patients^11^. The clusters of physicians in these networks often do not coincide with institutional boundaries but instead extend across them^12^. The literature on geographic variations literature in healthcare costs and outcomes was launched by Wenberg and Gittelsohn^13^, and has since become the central empirical argument for the inefficiency of the health care system in the Unites States. Because geography places constraints on patient-sharing relationships of physicians, a principled way to assess the impact of distance on intensity of connections in these networks might lead to a more complete examination of the sources of variability in provision of healthcare. Although we do not pursue this application here, the methods we introduce could also be used to address the role of geography also in healthcare delivery.

Because traditional surveys are resource intensive and scale poorly, mobile phone data, or more specifically call detail records (CDRs), have emerged as an alternative for inferring the structure of underlying interpersonal interactions^14–16^. Although user interactions on the mobile phone network are not limited by geography, users themselves are subject to spatial constraints that restrict the locations they may frequent and therefore influence their overall interpersonal and mobile phone communication patterns. For example, an individual-level analysis^17^ demonstrated a relationship between spatial configuration of offices and social connections among employees, and overlap of geographical space and information flow network is discussed^18^ from a perspective of the spread of knowledge and innovations. The effect of geographic restrictions may differ for locations in different regions. For example, in Belgium^19,20^, cell phone users communicate mostly within language-specific network communities^21^ of French and Flemish speakers. In addition, contact patterns among individuals that can result in disease transmission have also been shown to be location-specific^22^. Potential overlap of the geographical and social networks on the topological level has also been explored. The connection between local network topology and tie strength was found to be consistent with the so-called weak-ties hypothesis^14,23^. However, geographical and network centrality were not found to be related^24^.

In this study, we investigate the impact of spatial distance on cell phone communication, which is quantified as the number of calls between two counties, using a statistical approach. Our choice of model is guided by the observation that the intensity of communication among groups of people tends to decay with geographical distance; furthermore, the rate of decay in intensity appears to differ between short and longer distances. Failure to recognize this feature would result in an over-simplified model, biased estimates, and unsatisfactory predictions^25^. To incorporate this feature, we allow for the existence of a change point in the relationship between communication intensity and spatial distance.

As the structure of electronic communication, mobility, travel, and in-person social interactions are all related, we make use of existing methods and models in these areas. Some of the most widely studied models in these fields are the gravity model^19,26–29^, the radiation model^25^, and the rank-based friendship model^30^. Both the radiation model and the rank-based friendship model make explicit mechanistic assumptions regarding the effect of distance and population sizes, and these models focus on prediction. The gravity model is simpler and ignores the geographical distribution of the population; it uses only the source and destination population sizes and the spatial distance between them. Here we extend the gravity model by relaxing the assumption of a constant fixed decay rate in distance. As has been noted by Simini et al.^25^, the unsatisfactory performance of the gravity model compared with the radiation model for prediction has been mainly due to the assumption of an identical decay rate for all distances. We therefore incorporate the potential for heterogeneity of distance effects into our model; and we also provide an estimate and a confidence interval for the change point–that is the distance at which slope changes.

## Results

### Data

**Figure 1:**
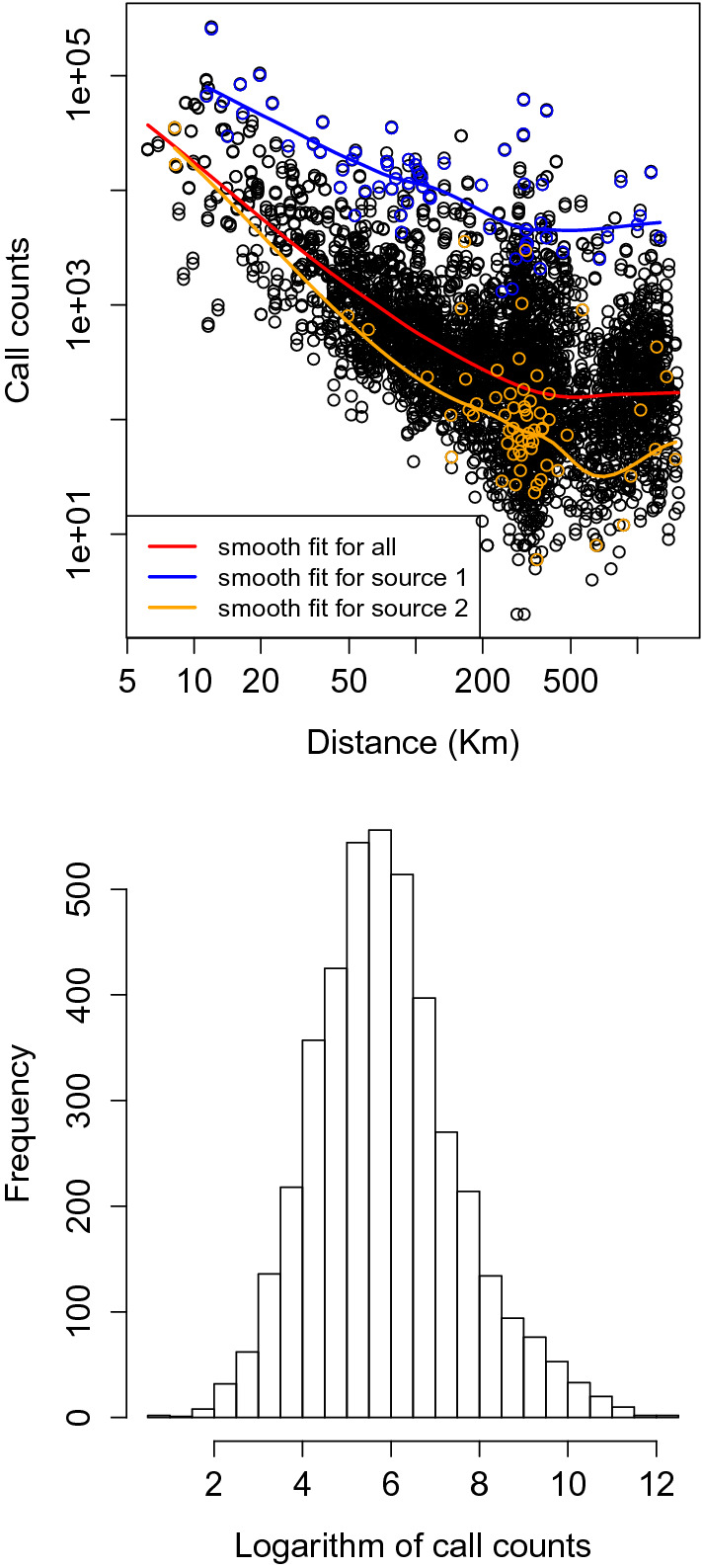
Top: scatter plot of natural log number of calls v.s. distances; bottom: histogram of natural log number of calls.

We aggregated the dataset in two ways. First, we aggregated the daily call counts over the 3-month period, resulting in a single call count for each distinct pair of users. We distinguish between the caller and the receiver; hence, the count for each call between each pair is directed. Second, we aggregated the data from the level of individuals to the level of counties; the resulting dataset describes communication intensity for calls among the counties. There were records for a total of 2,511,035 users; 359,759 of them resided in the largest county and 136, in the smallest. The number of calls from one county to another ranged from 0 to 266,199 with 21,016,548 calls in total. There were 2,646 distinct zip codes nested within 427 counties. The geographical location of each county was calculated by first identifying the latitude and longitude of each zip code centroid and then taking the mean of the these coordinates over all zip codes that were nested within a given county. For each county we thus obtained the number of resident users; and for each pair of counties, we obtained the spatial distance between them and the number of calls made and received by users in those counties over the 3-month period. As discussed in the section, Computational complexity, we reduce computational burden by selecting a subset of data that arose from 65 counties with the greatest numbers of users; in this subset, the number of calls ranged from 7,879 to 359,759. The corresponding call counts between pairs of counties ranged from 2 to 266,226. Multiple calls between any pair of users were included as one number in the call count. Figure 1 demonstrates the decay in intensity with distance as well as the distribution of number of calls; the log transformed call numbers appear to be roughly normal in distribution.

The distance is calculated at a coarser level (county) rather than at the zipcode level to protect user privacy; call counts between zipcodes might reveal user identity, especially between those for which the number of users and calls is small. We also note that although our analysis is of the locations of calls (not residences of callers), using a larger geographical unit will make these more likely to be the same, and perhaps thereby add to the interpretability of the analyses. We comment on this issue in the discussion.

### Gravity model and our extension

Analyses of the data described above is based on the gravity model. Adapting the notation from^26^, this model can be written as

1 $$\begin{aligned} G_{ij}=K \dfrac{m_i n_j}{d_{ij}^2}, \end{aligned}$$

where $G_{ij}$ specifies the communication intensity from source location *i* to destination location *j*, *K* is a constant, $m_i$ is the population of the source location *i*, $n_j$ is the population of the destination location *j*, and $d_{ij}$ is the distance between source *i* and destination *j*.

A related article^25^ provided an extension to this model:

2 $$\begin{aligned} G_{ij}= \dfrac{m_i^{\alpha } n_j^{\beta }}{f(d_{ij})}, \end{aligned}$$

where $f(\cdot )$ is a function that specifies the decay of $G_{ij}$ with distance $d_{ij}$, and it is usually specified as $d_{ij}^{\gamma }$. Here, we adopt the following form of the model:

3 $$\begin{aligned} G_{ij}= K \dfrac{m_i^{\alpha } n_j^{\beta }}{d_{ij}^{\gamma }}. \end{aligned}$$

Taking the logarithm of this expression yields

4 $$\begin{aligned} \log (G_{ij})= \log (K) + \alpha \log (m_i) + \beta \log (n_j) - \gamma \log (d_{ij}). \end{aligned}$$

### Inclusion of change points

We further extend the gravity model shown in Eq. (4) as follows:

5 $$\begin{aligned} \begin{aligned} Y_{ij}&= \mu + \beta _1 \log (n_i) + \beta _2 \log (n_j) + \beta _{3,i} \log (d_{ij}) + \beta _{4,i} (\log (d_{ij})-\theta _{i})_+ + \epsilon _{ij},\\&i,j=1,\ldots ,S; j \ne i, \end{aligned} \end{aligned}$$

where $n_i$ and $n_j$ are the number of users in county *i* and *j*; $d_{ij}$ is the distance between the two in kilometers; $Y_{ij}=g(G_{ij})$ and $g(\cdot )$ is a transformation function, in the gravity model, $g(\cdot )=\log (\cdot )$; $\mu$ is the intercept; $\theta _{i}$ represents the location of the change point measured on the logarithmic scale for communication initiated from location *i*; $\beta _{3,i}$ represents the distance effect before change point $\theta _{i}$; $\beta _{4,i}$ specifies the difference of distance effect before and after the change point; and *S* is the number of locations under consideration. When $\beta _{4,i}=0$, the difference is 0, i.e. the rate of decay does not change over the observed range. We denote the size of the population at location *i* as $n_i$ and refer to the model with $\beta _{4,i}$ as the *full model* and the model that sets $\beta _{4,i}$ to 0 as the *reduced model*. By definition, $(d_{ij}-\theta _{i})_+=(d_{ij}-\theta _{i}) I (d_{ij}>\theta _{i})$, where $I(\cdot )$ is the indicator function. It takes value 0 before the change point $\theta _{i}$ and $d_{ij}-\theta _{i}$ after the change point. We assume that $\epsilon _{ij} {\mathop {\sim }\limits ^{iid}} N(0, \sigma ^2)$. This formulation provides a straightforward way to compare the two nested models with regard to the effect of distance effect; the reduced model has the constraint $\beta _{4,i}=0$. In this formulation, model selection only involves variable selection; we perform the latter using LASSO^31^ . We also estimate $\theta _i$ and quantify its uncertainty as described in Methods below. We note that the above formulation assumes that the full and nested models share the same intercept and population size effects—an assumption that might not hold in practice. To address this concern, we consider two distinct settings, *case I*, which refers to the setting where the assumption holds, and *case II*, where it does not. For the latter, we extend the model by allowing different intercepts and population size effects for models with and without change points. In Methods, we describe how inference on this model is achieved.

## Analysis of call records data

As illustrated by the scatter plot in Fig. 1, the relationship between natural log of call counts and natural log of geographical distances appears to follow a linear relationship both before or after the break point. We also note that Fig.  1 is consistent with our assumptions of continuous calling intensity and normality of natural log of the number of calls. We used the preliminary binary assignments of change points based on BIC in a simple linear regression to assess whether there is variability across counties in intercepts and population size effects. Both models with only main effects (indicator variable of group assignments, log population sizes, log distance-before/after change point) and those with main effects and interaction terms showed evidence (p value < 0.05) of such variability. Hence we applied the method described below (in the Simulation study section) for the analysis of the cell phone data. The variability in intercepts and population size effects is true both for the general population from all 427 counties and for the user subpopulation we described above.

**Figure 2:**
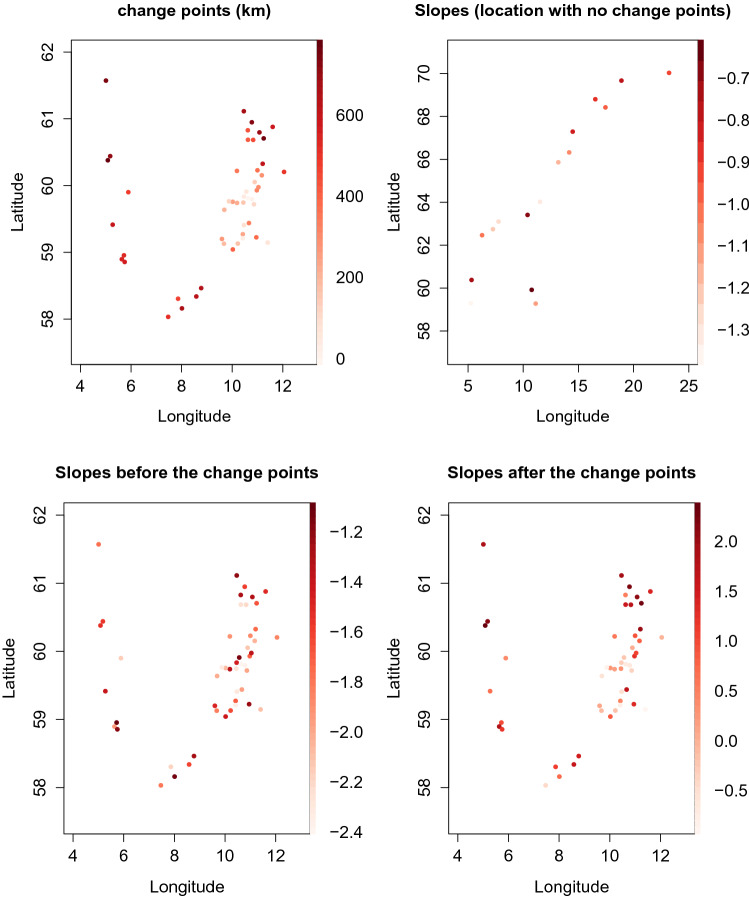
Estimated parameter values shown based on the geographical location (longitude and latitude) of the source locations. Top left: slope estimates for locations without change points; top right: log distance of the estimated change points for locations with change points; bottom: slopes estimates before and after change points for different locations.

**Figure 3:**
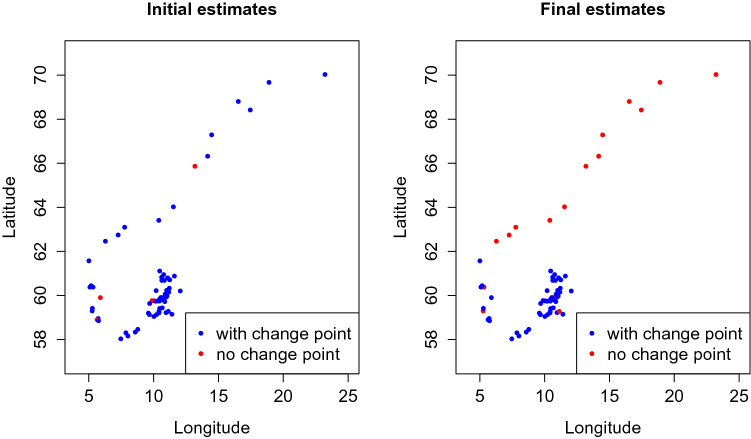
Initial and final estimates of the existence of the change points displayed based on the geographical location of the source county.

**Figure 4:**
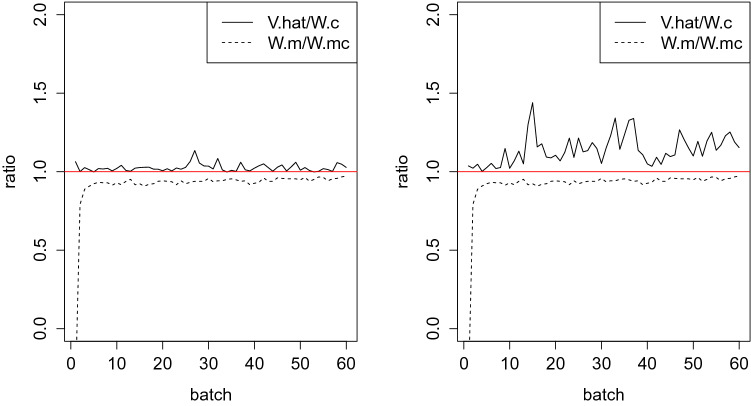
$\text {PSRF}_2$ approaches 1 very quickly and $\text {PSRF}_1$ fluctuates below 1.5. Left: diagnostic graph based on intercept estimates; right: diagnostic graph based on $\sigma ^2$; solid line: $\text {PSRF}_1$, dashed line: $\text {PSRF}_2$.

In the analysis of call records (Figs. 2 and 3), we note that the slopes for source locations in the northeast appear to be less steep; that slopes near the capital city, where the population is dense, are more likely to have change points No such patterns were observed for slopes of other locations, either before or after the change points. Model estimates revealed that locations with no change point tended to be in the north while those with change points were concentrated in the south around the capital area. For diagnosis on convergence, Fig. 4 shows a trend of $\text {PSRF}_2$ approaching 1 very quickly and a $\text {PSRF}_1$ fluctuating below 1.5, which is acceptable.

## Discussion

To analyze the decline in communication intensity with geographical distance, we extended the gravity model by allowing for change points in this relationship. We addressed the issue of the existence of change points for each source location and quantified associated uncertainty using a Bayesian model. We also provided estimates of the slopes before and after each change point. We investigated the geographical pattern of the existence of change points and noted differences in these patterns between rural and urban areas.

We apply our method to an anonymized dataset of call detail records, using the number of mobile phone calls in as the measure of communication intensity between a pair of counties. The outcomes are log-transformed counts; the regression model we specify treats the transformed outcomes as continuous—a choice that is most appropriate when the number of calls between two locations is large (Fig. 1). In settings with 0 or very small counts, one could consider alternative models (e.g., negative binomial) or the addition of an arbitrary small positive number to 0, although the latter approach can add bias^32,33^. In this setting, a negative binomial model might be a better fit, though the interpretation of the parameters is less straightforward. Using Bayesian methods in a setting where the data are assumed to be negative binomial distributed requires non-standard approaches even without inclusion of change points into models. Some research has provided useful tools for sequentially updating the parameters using Gibbs sampler by augmenting the posterior distribution with auxiliary parameters^34–36^. When the number of counts is large, the negative binomial approach may not be computationally feasible; fitting negative binomial outcomes in Bayesian LASSO needs further investigation. One possible direction is to extend the methods based on the conditional normal distribution^36^ by transforming the variance matrix so that normal-distribution based LASSO method can be employed.

Another extension of our method would allow for aggregation of results across different subsamples; currently the number of locations we can analyze is limited by computational capacity. Developing a method to obtain consistent results from different overlapping sets of nodes, perhaps in a meta-analysis framework, would alleviate the computational concerns, but is challenging. Some potentially useful approaches are provided^37–40^. In particular, the stability selection^41^ may be used to assess the properties of the meta-analytic results. An example of the use of LASSO in analyses that combine across subsamples arose from analyses intended to discover adverse drug reactions^42^. Another potentially useful approach is the use of path of partial posteriors^43^. In this approach, the resampling procedure resembles the bootstrap, but with smaller resampling sizes. Because standard bootstrapping of the LASSO estimator of the regression parameter for variance inference is known to yield inconsistent estimates^44,45^, modified bootstrapping must be used^46^. Nonetheless, Bayesian LASSO procedures provide straightforward and valid estimates for standard errors.

The findings from our analysis of mobile phone communication intensity illustrate how such information might be used. For example, should such communication networks prove to be accurate proxies for contact networks, such analyses might help guide the design of cluster randomized trials for infectious disease. Randomized trials ideally enroll participants in a way that minimizes the extent to which the treatment assignment of one subject affects the outcome of another. For interventions in which such interference occurs at the individual but not the cluster level (e.g., through contacts among randomized subjects), cluster randomization can be useful^47^. Clusters may be comprised of participants in the same geographical location, institution (e.g. school) or administrative unit (village). Cell phone data could potentially aid in the identification of appropriate clusters by providing information about the probability of interference. When mixing across clusters cannot be eliminated, identification of treatment effects requires modeling of the mixing process^48^. The impact of interference across randomized units on power of a clinical trial to detect effects of an intervention in preventing spread of infectious disease is investigated^49,50^. As geographical distance is likely to affect contact networks, knowing the relationship between communication and distance may be useful not only for identification of clusters, but also for aiding in development of appropriate mixing models.

## Methods

To estimate the parameter of interest, $\theta _i$, and quantify its uncertainty we employ a Metropolis Hastings algorithm in Bayesian framework. We consider a Metropolis sampling block for $\theta _i$ and a Bayesian LASSO block dealing with β_4,i_. To allow different intercepts and population size effects for models with and without change points, we employ a Reversible Jump Monte Marlo Markov Chain algorithm. To implement it, we chooose (RJMCMC) option in the blasso function in R package monomvn. We use the default non-informative priors for unknown parameters in both simulation and data analysis. This approach allows for statistical inference using Bayesian LASSO. RJMCMC is a general version of the Metropolis-Hastings algorithm^51^, which allows transitions between models of different dimensions. In our setting, the RJMCMC sampling procedure allows changes in the model based on the variable selection results from the previous iteration; the intercept and population size effects are modeled separately for the two models. We provide details below.

### Sampling algorithm

#### Initial values

To speed up convergence of RJMCMC algorithm and prevent it from converging to a local mode, we calculate a set of crude initial values for all the parameters as follows:

1. Search through a grid over the distance range of location *i* for $\theta _i$ and choose the grid point that maximizes the likelihood function of the crude full model $\varvec{\theta ^{(0)}}$.
2. For case I, the preliminary values for the parameters are obtained by linear regression treating the change points as known. Substituting in the value of $\varvec{\theta ^{(0)}}$ from Step 1 leads to crude parameter estimates $\mu ^{(0)}$, $\varvec{\beta ^{(0)}} \equiv {(\beta _1^{(0)}, \beta _2^{(0)}, {\varvec{\beta _{3}^{(0)}}}^T, {\varvec{\beta _{4}^{(0)}}}^T)}^T$ and $\sigma ^{2}_{(0)}$. For case II, we fit two models for each source location: Model 1 has a change point at $\varvec{\theta ^{(0)}}$ estimated in Step 1 and Model 2 has no change point. We then assign $\eta _{i}^{(0)}=1$ if Model 1 has a lower BIC than Model 2, and assign $\eta _{i}^{(0)}=0$ otherwise. We use BIC to account for the fact that Model 1 has more parameters than Model 2. Based on $\varvec{\eta ^{(0)}} \equiv (\eta _{1}^{(0)},\eta _{2}^{(0)},\ldots ,\eta _{S}^{(0)})^T$, we create a new corresponding model matrix, removing the column of $\beta _{4,i}$ if $\eta _{i}^{(0)}=0$, and obtain the crude parameter estimates $\varvec{\mu ^{(0)}, \beta ^{(0)}}$ and $\sigma ^{2}_{(0)}$ from linear regression. For cases where $\eta _{i}^{(0)}=0$, we assign $\beta _{4,i} = 0$.

#### Metropolis block and Bayesian LASSO

*Case I: Assuming same intercept and population size effects across all source locations* With Bayesian LASSO, the model is specified as

6 $$\begin{aligned} \begin{aligned} Y_{ij}&= \mu + \beta _1 \log (n_i) + \beta _2 \log (n_j) + \beta _{3,i} \log (d_{ij}) + \beta _{4,i} (\log (d_{ij})-\theta _{i})_+ + \epsilon _{ij},\\&\theta _i \in (\min \limits _{j} \log (d_{ij}), \max \limits _{j} \log (d_{ij})), i,j=1,\ldots ,S, j \ne i, \end{aligned} \end{aligned}$$

which can be written as $\varvec{Y=}\mu \varvec{1}+\varvec{X\beta +\epsilon }$ using matrix notation. $\mu$ is not included in the Bayesian LASSO penalty term^52^; $\varvec{1}$ is the vector of 1s; $\varvec{X}$ is the model matrix consisting of logarithmic population sizes and distances, and $\varvec{\beta }$ is the vector of $\beta$s.

In general, LASSO^31^ solves an unconstrained optimization problem subject to a given bound on the $L_1$ norm of the parameter vector that is equivalent to

7 $$\begin{aligned} \min \limits _{\varvec{\beta }} \varvec{(\tilde{Y}-X\beta )^T(\tilde{Y}-X\beta )}+\lambda \sum \limits _{j=1}^{p} |\beta _j|, \end{aligned}$$

where $\varvec{\tilde{Y}=Y}-\mu \varvec{1}$ is the centered outcome vector; *p* is the number of parameters after excluding the intercept. In the Bayesian setting, solution to Eq. (7) provides the posterior mode estimates when $\beta _j$ has i.i.d. double exponential priors. Conditional double exponential priors are used in the formulation to avoid multiple modes^52^. They can be expressed hierarchically as

8 $$\begin{aligned} & \varvec{Y}|\mu , \varvec{X}, \varvec{\beta}, {\sigma}^{2} \sim N(\mu \varvec{1}+\varvec{X} \varvec{\beta}, \sigma^{2} \varvec{I}), \\ & \varvec{\beta}|\tau_{1}^{2}, \ldots , {\tau}_{p}^{2}, {\sigma}^{2} \sim N(\varvec{0}, \sigma^{2} {\varvec{D}_{\varvec{r}}}), \text{where} {\varvec{D}_{\varvec{r}}}=\text{diag}({\tau}_{1}^{2}, \ldots , {\tau}_{p}^{2}), \\ & \sigma ^2, {\tau}_{1}^{2}, \ldots , \tau_{p}^{2} \sim \pi (\sigma^{2}) d\sigma^{2} \prod_{j=1}^{p} \frac{\lambda^{2}}{2} e^{-\lambda^{2} \tau_{j^2/2}} d\tau_{j^2}, \sigma^{2}, \tau_{1}^{2}, \ldots , \tau_{p}^{2} >0. \end{aligned}$$

The entire sampling procedure is available using function blasso in R package monomvn with the option for RJMCMC specified as False. To incorporate a Metropolis block for change point estimation, we alternate between the Metropolis and Bayesian LASSO blocks. Validity of this approach is established by regarding it as two components of a Gibbs sampling algorithm^53^. In summary, conditional on change points, our inferential problem becomes one of a variable selection; conditional on other parameters, change point sampling is a straightforward application of a Metropolis algorithm.

Thus after obtaining the initial values $\varvec{\mu ^{(0)}, \beta ^{(0)}, \theta ^{(0)}}$ and $\sigma ^{2}_{(0)}$, we proceed as follows:

1. At iteration *t* for each source location *i*, update change point $\theta _{i}^{(t+1)}$ using Metropolis algorithm with a normal proposal $N(\theta _i^{(t)}, \sigma ^2_{\theta })$. The range of $\theta _i$ is determined empirically from data, i.e., the posterior likelihood of $\theta _i$ has an indicator function term in the product that is 0 if the proposed $\theta _{i}^{(t+1)}$ is out of the observed empirical log-distance range, thereby assuring that any out-of-range proposal will be rejected.
2. For each location *i*, if there are fewer than 5% of data points on either side of $\theta _{i}^{(t+1)}$ for the subset of data, i.e., $\varvec{Y_{i}}$, we consider it to be on the boundary, specify $\beta _{4,i}^{(t+1)}=0$, and remove it from the model in the next estimation step. We denote the number of locations belonging to the boundary sets as $b^{(t+1)}$.
3. Create the corresponding $S(S-1) \times (2+2S-b^{(t+1)})$ covariate matrix (intercept column is not included) based on $\varvec{\theta ^{(t+1)}}$. Together with the data, $\varvec{\beta ^{(t)}}$ (after $\beta _{4,i}^{(t+1)}=0$ are removed), $\sigma ^{(t) 2}$ and $\lambda ^{(t)}$, input the covariate matrix into the blasso function for *h* iterations (2 or more). The output intercept is $\mu ^{(t+1)}$. From the output we also get $\varvec{\beta ^{(t+1)}}$ ($\beta _{4,i}^{(t+1)}=0$ are put back), $\sigma ^{(t+1) 2}$ and $\lambda ^{(t+1)}$.
4. Repeat steps 1-3 until convergence (see below).

*Case II: Allowing different intercepts and population size effects for models with and without change points.*

When there is evidence of the presence of change points, we estimate these parameters separately in two different models. In this case, estimates of intercepts and population size effects depend on the set of source locations whose data contribute to the estimation in any given iteration. We denote the mean model as $\varvec{\eta ^{(t)}}$ for iteration *t* to maintain consistency with the notation we introduced earlier.

As mentioned above, estimation makes use of the Reversible Jump MCMC option in the blasso function. In our setting, different models imply different specification of zeros in $\varvec{\beta _4^{(t)}}$ , and are characterized by $\varvec{\eta ^{(t)}}$, where $\eta _i^{(t)}=I(\beta _{4,i}^{(t)}>0)$.

RJMCMC is a general version of the Metropolis-Hastings algorithm^51^, which allows transitions between different states or models of different dimensions. A thorough review of RJMCMC with more recent comments can be found in a review article^54^.

Use of RJMCMC yields the following sampling scheme:

1. The first two steps are the same as in case I: At iteration *t*, for each source location *i*, update change point $\theta _{i}^{(t+1)}$ using Metropolis algorithm with a normal proposal $N(\theta _i^{(t)}, \sigma ^2_{\theta })$. For each location *i*, if there are fewer than 5% of data points on either side of $\theta _{i}^{(t+1)}$ for $\varvec{Y_{i}}$, we specify $\beta _{4,i}^{(t+1)}=0$ and remove it from the model in the next estimation step.
2. Conditional on $\varvec{\theta ^{(t+1)}}$, create the $s(s-1) \times (5+2s-b^{(t+1)})$ covariate matrix (intercept column is not included). Data from each source location contribute to their own group’s estimation of intercept and population size effects, which depends on $\varvec{\eta _i^{(t)}}$. All data and parameter values from the previous iteration *t* (including $\sigma ^{(t) 2}$ and $\lambda ^{(t)}$) are used in the blasso function with RJMCMC for 3 iterations. 3 is the minimum number of iterations to avoid the situation in which zeros in the previous iteration are carried forward.
3. From Step 2 we get the updated $\varvec{\beta ^{(t+1)}}, \sigma ^{(t+1) 2}, \mu ^{(t+1)}$ and $\lambda ^{(t+1)}$. Now update the $\varvec{\eta ^{(t+1)}}$: $\eta _i^{(t+1)}=1$ if $\beta _{4,i}^{(t+1)}>0$; otherwise 0.
4. Repeat steps 1-3 until convergence.

### Diagnostics for assessment of convergence

The usual diagnostic framework for Bayesian LASSO^55–57^ includes trace plots for different chains and calculation of the *Potential Scale Reduction Factor* (PSRF). Diagnostics for RJMCMC can be developed by extending that framework to include within-model and between-model variations in the parameters.

We make use of Castello and Zimmerman^58^, which defines two PSRFs in the assessment. For a chosen parameter, $\text {PSRF}_1$ is the ratio between total variation $\widehat{V}$ and variation within chains $W_c$; $\text {PSRF}_2$ is the ratio between variation within models $W_m$ and variation within models and chains $W_{m}W_{c}$. $\widehat{V}, W_c, W_m$ and $W_{m}W_{c}$ are defined as follows:

9 $$\begin{aligned} \begin{aligned} \widehat{V}(\theta )&= \dfrac{1}{CT-1} \sum \limits _{c=1}^{C} \sum \limits _{m=1}^{M} \sum \limits _{r=1}^{R_{cm}} (\theta _{cm}^r-\overline{\theta _{..}}^.)^2, \\ W_c(\theta )&= \dfrac{1}{C(T-1)} \sum \limits _{c=1}^{C} \sum \limits _{m=1}^{M} \sum \limits _{r=1}^{R_{cm}} (\theta _{cm}^r-\overline{\theta _{c.}}^.)^2,\\ W_m(\theta )&= \dfrac{1}{CT-M} \sum \limits _{c=1}^{C} \sum \limits _{m=1}^{M} \sum \limits _{r=1}^{R_{cm}} (\theta _{cm}^r-\overline{\theta _{.m}}^.)^2,\\ W_{m}W_{c}(\theta )&= \dfrac{1}{C(T-M)} \sum \limits _{c=1}^{C} \sum \limits _{m=1}^{M} \sum \limits _{r=1}^{R_{cm}} (\theta _{cm}^r-\overline{\theta _{cm}}^.)^2,\\ \end{aligned} \end{aligned}$$

where $\theta _{cm}^r, \overline{\theta _{..}}^., \overline{\theta _{c.}}^., \overline{\theta _{.m}}^.$ and $\overline{\theta _{cm}}^.$ are the *r*th appearance of $\theta$ in model *m* chain *c*, mean $\theta$ across all models and chains, mean $\theta$ within chain *c* across all models in that chain, mean $\theta$ within model m across all chains, mean $\theta$ within chain *c* and model *m*, respectively. $R_{cm}$ is number of $\theta$ in chain *c* model *m*. *C* and *M* are the number of chains and distinct models, respectively.

We follow the strategy provided by Castello and Zimmerman^58^ to assess convergence and, for simplicity, illustrate this approach by considering a scalar. We choose $\sigma ^2$, the variance of the error terms, for this illustration, as its interpretation remains the same across the models. Each chain is divided into batches of equal length. A sequence of $\text {PSRF}_1$ and $\text {PSRF}_2$ is calculated for each batch. A desirable result is that the two quantities move toward 1 as the iteration proceeds. In the simulation study below, we illustrate the use of diagnostic graphs for evaluating convergence; further details on this subject can be found in Brooks and Giudici^59^.

### Interpretation

Under the assumption that intercept and population size effects are identical across source locations, we obtain a sample of $\beta _{4,i}$ as well as its 95% credible interval rather than an estimate of the probability that each source location has a change point. Intervals that do not cover 0 imply the presence of a change point by providing evidence against the null hypothesis that the difference of the two slopes is zero. Approaches that allow variability in intercepts and population size effects yield a sample of models and their corresponding parameter estimates. For prediction, we make use of the models that RJMCMC has sampled in the estimation process; the estimated mean for predicted outcomes is a weighted average of the predicted outcomes of all models.

### Computational complexity

Because of the computational burden of these methods, we consider an analysis of a subset of data. Simulation studies (Fig. 6 in Appendix) show that computation time for the Bayesian LASSO function blasso increases sharply as the number of locations increases. We note that the size of the covariate matrix increases at $O(S^3)$ where *S* specifies the number of locations. It has been showed that for the least angle regression formulation of the problem, the computational complexity is $O(m^3+m^2 n)$^60^, where *m* is the number of features and *n* is the number of the outcomes. In our setting, the situation is even more challenging in that the number of outcomes grows quadratically with *S*, which renders the overall computational complexity to be $O(S^4)$.

### Simulation study

We conducted the following simulations to assess the performance of our models compared with naïve approaches as well as to check the effect of the tuning parameter $\sigma ^2_{\theta }$. The values of the parameters in the data generation process were selected to be the estimates from the preliminary data analysis using $\sigma ^2_{\theta }=0.03$. The observed geographical distances between counties were used. We assessed the performance of the gravity model, the naïve fit based on BIC and grid search, and the Bayesian LASSO model on scenarios with low (0.30), medium (0.38) and high (0.45) error variances ($\sigma ^2$). The medium value was selected to match the estimates from the preliminary analyses. For each scenario, we simulated 2 data sets and applied our algorithm with 4 chains. We also evaluated the effect of the tuning parameter $\sigma ^2_{\theta }$ for the Metropolis algorithm by specifying a series of different values for it: 0.015, 0.02, 0.025, 0.03, 0.04, 0.05, 0.06, 0.08, 0.1, 0.12, 0.15, 0.2, 0.25, 0.3, 0.4, 0.6. The diagnostic graphs in Appendix show that convergence was generally achieved. We assessed the model fit and the effect of the tuning parameter based on the prediction error (PE), which is defined as follows:

10 $$\begin{aligned} PE(L) = \dfrac{1}{M} \sum (y_{new}-\widehat{y_{new}})^2, \end{aligned}$$

where *L* is the model, *M* is the number of data points, $y_{new}$ is the observed outcome in the test dataset, $\widehat{y_{new}}$ is the fitted value using model estimated on the old dataset.

One hundred new datasets were generated using the same covariates and parameters for each variance category. The findings are shown in Table 1.

**Table 1:** Prediction error of the gravity model, the naïve fit based on BIC and grid search, and the Bayesian LASSO model in scenarios with low (0.30), medium (0.38) and high (0.45) error variances ($\sigma ^2$) (2 trials each).

	Variance of error term $\sigma ^2$					
	0.30		0.38		0.45	
Gravity model						
	0.807	0.807	0.887	0.887	0.956	0.956
Crude model based on BIC						
	0.331	0.327	0.412	0.435	0.485	0.486
Bayesian LASSO with change points						
$$\sigma ^2_{\theta }$$						
0.015	0.321	0.329	0.403	0.411	0.479	0.486
0.020	0.322	0.329	0.405	0.413	0.479	0.487
0.025	0.319	0.329	0.404	0.411	0.479	0.486
0.030	0.321	0.326	0.407	0.411	0.479	0.485
0.040	0.318	0.323	0.409	0.409	0.481	0.486
0.050	0.317	0.323	0.411	0.411	0.480	0.487
0.060	0.318	0.321	0.411	0.411	0.481	0.487
0.080	0.318	0.321	0.411	0.410	0.482	0.487
0.100	0.318	0.320	0.411	0.409	0.482	0.488
0.120	0.320	0.320	0.410	0.412	0.481	0.485
0.150	0.319	0.320	0.411	0.414	0.486	0.487
0.200	0.321	0.319	0.413	0.414	0.486	0.490
0.250	0.321	0.320	0.415	0.416	0.489	0.488
0.300	0.320	0.321	0.413	0.417	0.490	0.489
0.400	0.321	0.321	0.417	0.420	0.496	0.489
0.600	0.325	0.322	0.414	0.419	0.494	0.489

**Figure 5:**
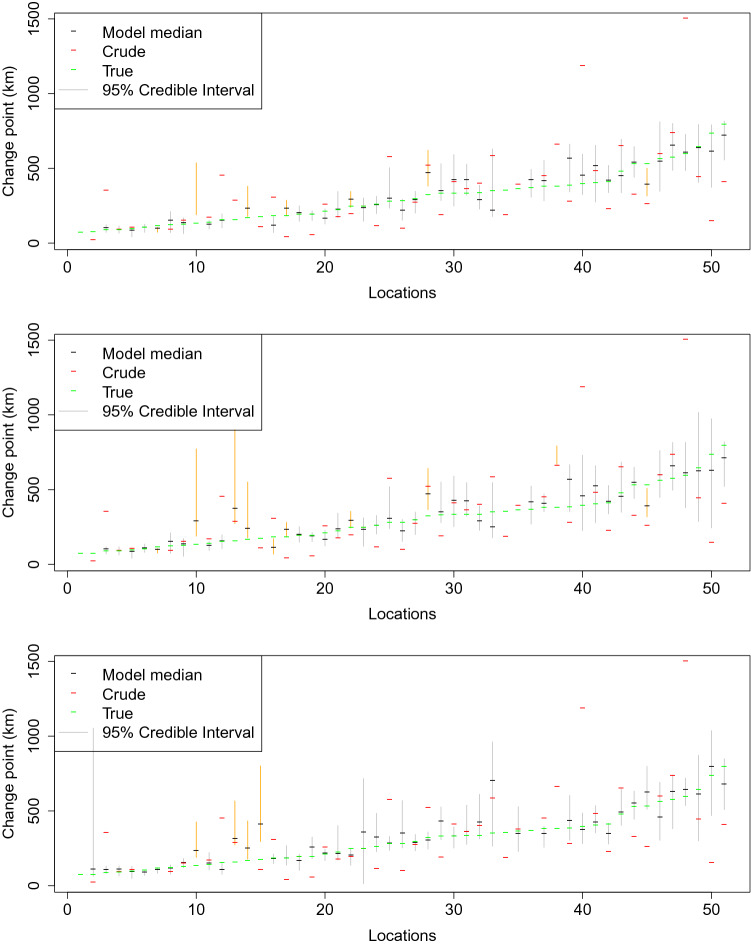
Estimated 95% credible intervals of change point $\theta _{i}$ (when true change points exist) under low (top), medium (middle) and high (bottom) error variance $\sigma ^2$ with tuning parameter $\sigma ^2_{\theta }=0.2$; orange color of the 95% credible interval indicates that the true value is not covered; if no 95% credible interval is shown, then none is available, i.e., estimates are from the model without change points. Locations have been ordered from left to right based on the true locations of the change points.

**Figure 6:**
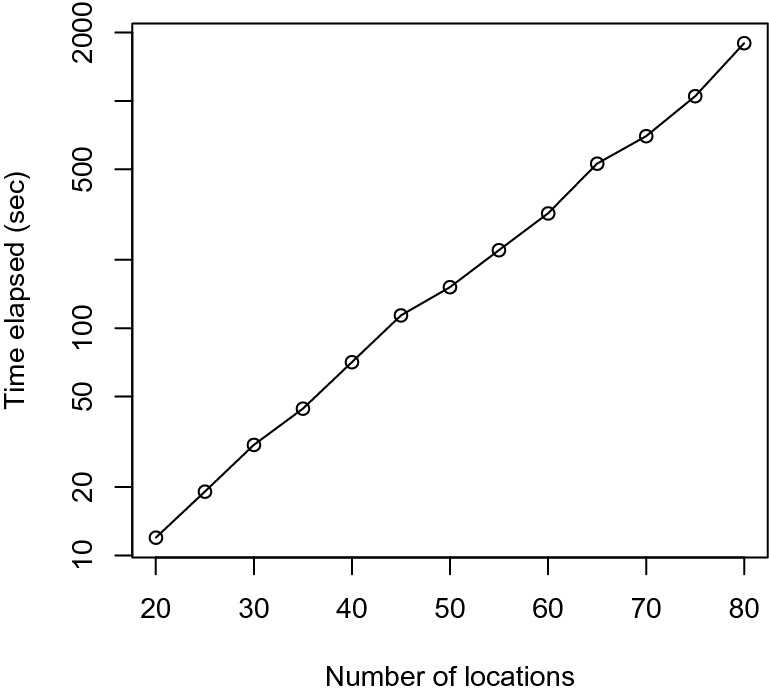
Runtime estimated for 50 iterations versus number of locations in the simulation. Note that the vertical axis is on logarithmic scale.

As expected, estimates based both on BIC and Bayesian LASSO performed better than those of the gravity model with respect to prediction error in low, medium, and high error variances. The choice of tuning parameter had little effect; use of 0.2 in data analysis appears reasonable as this choice leads to a mean acceptance rate for the Metropolis algorithm on change points in the range of 20–25%^57^, as shown in Table 2. The 95% credible interval coverages for change points, as shown in Fig. 5 and Table 3, also reached high values at tuning parameter 0.2. The crude model based on BIC and Bayesian LASSO estimates are comparable. This is demonstrated in Fig. 5, which shows the crude estimates and Bayesian LASSO estimates to be similar. An advantage of the latter however is its ability to provide interval estimates on the change points and its smaller number of required parameters; Fig. 5 provides the 95% credible interval. These results imply that predictive power was not reduced because of the estimation of location of change points. Bayesian LASSO does require greater computation time: Computation time for 15,000 iterations takes around 9–10 h, whereas the BIC approach requires only a few minutes. For further information about runtime from simulation studies, see Fig. 6.

**Table 2:** Mean acceptance rate for Metropolis algorithm on change points in scenarios with low (0.30), medium (0.38) and high (0.45) error variances ($\sigma ^2$) (2 trials each).

$\sigma ^2_{\theta }$	Variance of the error terms $\sigma ^2$					
	0.30		0.38		0.45	
0.015	0.544	0.543	0.550	0.546	0.561	0.561
0.020	0.518	0.516	0.522	0.521	0.527	0.534
0.025	0.493	0.495	0.501	0.498	0.509	0.509
0.030	0.471	0.471	0.482	0.470	0.495	0.486
0.040	0.442	0.433	0.443	0.447	0.463	0.455
0.050	0.410	0.412	0.420	0.417	0.439	0.433
0.060	0.388	0.388	0.394	0.395	0.410	0.415
0.080	0.341	0.346	0.359	0.354	0.373	0.370
0.100	0.300	0.313	0.322	0.321	0.338	0.333
0.120	0.277	0.283	0.292	0.292	0.308	0.304
0.150	0.243	0.245	0.254	0.260	0.273	0.270
0.200	0.194	0.198	0.208	0.207	0.222	0.227
0.250	0.166	0.167	0.179	0.173	0.185	0.189
0.300	0.143	0.144	0.154	0.156	0.161	0.162
0.400	0.110	0.110	0.122	0.118	0.127	0.129
0.600	0.075	0.073	0.083	0.084	0.090	0.088

**Table 3:** 95% credible interval coverage for change points in scenarios with low (0.30), medium (0.38) and high (0.45) error variances ($\sigma ^2$) (2 trials each).

$\sigma ^2_{\theta }$	Variance of the error terms $\sigma ^2$					
	0.30		0.38		0.45	
0.015	0.585	0.585	0.523	0.492	0.462	0.492
0.020	0.631	0.615	0.554	0.523	0.508	0.569
0.025	0.662	0.631	0.554	0.523	0.585	0.600
0.030	0.677	0.677	0.615	0.554	0.600	0.631
0.040	0.723	0.723	0.662	0.631	0.615	0.615
0.050	0.754	0.692	0.692	0.692	0.677	0.646
0.060	0.754	0.708	0.692	0.692	0.646	0.646
0.080	0.754	0.754	0.677	0.738	0.692	0.677
0.100	0.785	0.785	0.692	0.754	0.723	0.692
0.120	0.769	0.815	0.708	0.738	0.769	0.708
0.150	0.769	0.815	0.723	0.738	0.738	0.692
0.200	0.785	0.877	0.723	0.738	0.769	0.677
0.250	0.785	0.862	0.662	0.738	0.754	0.708
0.300	0.769	0.862	0.708	0.738	0.738	0.692
0.400	0.769	0.862	0.677	0.708	0.708	0.708
0.600	0.785	0.831	0.708	0.738	0.692	0.708

## Data Availability

The data that support the findings of this study is available from Telenor and was obtained for this research by Dr. Onnela. Restrictions apply to the availability of these data, and so are not publicly available.

## Appendix: Discussion of model choices

In addition to the gravity model, other models to study the impact of spatial distance on communication intensity, such as the radiation model^25^, have been proposed. This model predicts commuting flux between locations, and the rank-based friendship model^30^, which ranks friendships based on the geographical distance between them. Both models reduce to Eq. (4) with certain constraints on their parameters or under certain assumptions. The radiation model^25^ uses the following specification

11 $$\begin{aligned} \langle T_{ij} \rangle = T_i \dfrac{m_i n_j}{(m_i + s_{ij})(m_i + n_j + s_{ij})}, \end{aligned}$$

where $\langle T_{ij} \rangle$ is the average commuting or mobility flux from location *i* to *j* (for simplicity, we denote average flux as $T_{ij}$ to keep the notation consistent), $T_i=\sum \nolimits _{j \ne i} T_{ij}$ is the total number of commuters from *i*, and $s_{ij}$ is the population living in the circle centered at the source with a radius of $r_{ij}$ (not including $m_i$). Adopting this notation,

12 $$\begin{aligned} T_{ij} = T_i \dfrac{m_i n_j}{(m_i + s_{ij})(m_i + n_j + s_{ij})}. \end{aligned}$$

Taking the logarithm yields,

13 $$\begin{aligned} \log (T_{ij}) = \log (T_i) + \log (m_i) + \log (n_j) - \log (m_i + s_{ij}) - \log (m_i + n_j + s_{ij}). \end{aligned}$$

In radiation model^25^, we note that Eq. (13) reduces to Eq. (4) with $\alpha +\beta =1$ and $\gamma =4$ when the population is uniformly distributed such that $m=n$ and $s_{ij} \approx m_i r_{ij}^2$. The model is mechanistic and has no parameter to fit.

The rank-based friendship model^30^ is formulated as follows. Let *u* and *v* be two individuals. Then define $\text {rank}_u (v)=|\{w: d(u,w) \leqslant d(u,v)\}|$, where *d*(*u*, *w*) is the distance between individual *u* and individual *w*. The probability of *u* and *v* being friends is modeled as

14 $$\begin{aligned} \text {Pr}[u \rightarrow u] \propto \dfrac{1}{\text {rank}_u(v)}. \end{aligned}$$

As $\text {rank}_u(v) \approx d(u,v)^2$ when the the population is uniformly distributed, Eq. (14) reduces to Eq. (4) with $m=n=1$ and $\gamma =2$.

Both the gravity and radiation models are based on strict assumptions of the underlying mechanism, which are hard to validate. The gravity model, which uses the same parameters for each pair of locations, implicitly assumes a homogeneous effect of distance for the intensity function. The radiation model addresses this issue by modeling the intrinsic heterogeneity of the geographical distribution of population by incorporating $s_{ij}$ in the model. However, subject to its strict assumption and ‘parameter-free’ property, it allows little room for other factors. The rank-based model deals with the heterogeneity by substituting distance with rank, which seems to have a similar role as the $s_{ij}$ in the radiation model. Thus the $\text {rank}$ function in Eq. (14) can be regarded as an implicit function of distance and population distribution. We can make Eq. (14) parametric by incorporating a parameter for the power of the rank. If the population is uniformly distributed across the area, this is equivalent to the gravity model with parameter $\gamma$ for the distance $r_{ij}$.

We note here that even though the rank-based approach sheds some lights on the question of interest, to move from the individual level to zip code or county level requires a completely different set of assumptions. Therefore, a rank-based gravity model cannot be seen as a simple extension of the rank-based friendship model.

## References

[1] Cho, E., Myers, S.A. & Leskovec, J. Friendship and mobility: user movement in location-based social networks. in *Proceedings of the 17th ACM SIGKDD international conference on Knowledge discovery and data mining*, 1082–1090 (ACM, 2011).

[2] Scellato, S., Noulas, A., Lambiotte, R. & Mascolo, C. Socio-spatial properties of online location-based social networks. in *Fifth International AAAI Conference on Weblogs and Social Media* (2011).

[3] Backstrom, L., Sun, E. & Marlow, C. Find me if you can: improving geographical prediction with social and spatial proximity. in *Proceedings of the 19th international conference on World wide web*, 61–70 (ACM, 2010).

[4] Eagle, N., Pentland, A.S. & Lazer, D. Mobile phone data for inferring social network structure. in *Social Computing, Behavioral Modeling, and Prediction*, 79–88 (Springer, 2008).

[5] Wang, D., Pedreschi, D., Song, C., Giannotti, F. & Barabasi, A.-L. Human mobility, social ties, and link prediction. in *Proceedings of the 17th ACM SIGKDD international conference on Knowledge discovery and data mining*, 1100–1108 (ACM, 2011).

[6] Hawelka B (2014). Geo-located twitter as proxy for global mobility patterns. Cartogr. Geogr. Inf. Sci..

[7] Eagle N, Pentland AS, Lazer D (2009). Inferring friendship network structure by using mobile phone data. Proc. Nat. Acad. Sci..

[8] Gregson S (2002). Sexual mixing patterns and sex-differentials in teenage exposure to hiv infection in rural zimbabwe. Lancet.

[9] Jones JH, Handcock MS (2003). An assessment of preferential attachment as a mechanism for human sexual network formation. Proc. R. Soc. Lond. B Biol. Sci..

[10] Helleringer S, Kohler H-P (2007). Sexual network structure and the spread of hiv in africa: evidence from Likoma island, Malawi. AIDS.

[11] Landon BE (2012). Variation in patient-sharing networks of physicians across the united states. JAMA.

[12] Landon BE (2013). Using administrative data to identify naturally occurring networks of physicians. Med. Care.

[13] Wennberg J, Gittelsohn A (1973). Small area variations in health care delivery: a population-based health information system can guide planning and regulatory decision-making. Science.

[14] Onnela J-P (2007). Structure and tie strengths in mobile communication networks. Proc. Nat. Acad. Sci..

[15] Buckee CO, Wesolowski A, Eagle NN, Hansen E, Snow RW (2013). Mobile phones and malaria: modeling human and parasite travel. Travel Med. Infect. Dis..

[16] Tatem AJ (2014). Integrating rapid risk mapping and mobile phone call record data for strategic malaria elimination planning. Malar. J..

[17] Sailer K, McCulloh I (2012). Social networks and spatial configuration-how office layouts drive social interaction. Soc. Netw..

[18] Ter Wal AL, Boschma RA (2009). Applying social network analysis in economic geography: framing some key analytic issues. Ann. Reg. Sci..

[19] Lambiotte R (2008). Geographical dispersal of mobile communication networks. Phys. A.

[20] Expert P, Evans TS, Blondel VD, Lambiotte R (2011). Uncovering space-independent communities in spatial networks. Proc. Nat. Acad. Sci..

[21] Porter MA, Onnela J-P, Mucha PJ (2009). Communities in networks. Not. AMS.

[22] Wang L, Wang Z, Zhang Y, Li X (2013). How human location-specific contact patterns impact spatial transmission between populations?. Sci. Rep..

[23] Granovetter MS (1973). The strength of weak ties. Am. J. Sociol..

[24] Onnela J-P, Arbesman S, González MC, Barabási A-L, Christakis NA (2011). Geographic constraints on social network groups. PLoS One.

[25] Simini F, González MC, Maritan A, Barabási A-L (2012). A universal model for mobility and migration patterns. Nature.

[26] Krings G, Calabrese F, Ratti C, Blondel VD (2009). Urban gravity: a model for inter-city telecommunication flows. J. Stat. Mech: Theory Exp..

[27] Balcan D (2009). Multiscale mobility networks and the spatial spreading of infectious diseases. Proc. Nat. Acad. Sci..

[28] Noulas A, Scellato S, Lambiotte R, Pontil M, Mascolo C (2012). A tale of many cities: universal patterns in human urban mobility. PLoS One.

[29] Csáji BC (2013). Exploring the mobility of mobile phone users. Phys. A.

[30] Liben-Nowell D, Novak J, Kumar R, Raghavan P, Tomkins A (2005). Geographic routing in social networks. Proc. Nat. Acad. Sci..

[31] Tibshirani R (1996). Regression shrinkage and selection via the lasso. J. R. Stat. Soc. Ser. B (Methodol.).

[32] Flowerdew R, Aitkin M (1982). A method of fitting the gravity model based on the poisson distribution. J. Region. Sci..

[33] Burger M, Van Oort F, Linders G-J (2009). On the specification of the gravity model of trade: zeros, excess zeros and zero-inflated estimation. Sp. Econ. Anal..

[34] Zhou, M., Li, L., Dunson, D. & Carin, L. Lognormal and gamma mixed negative binomial regression. in *Machine learning: proceedings of the International Conference. International Conference on Machine Learning*, vol. 2012, 1343 (NIH Public Access, 2012). PMC4180062 25279391

[35] Pillow JW, Scott JG (2012). Fully bayesian inference for neural models with negative-binomial spiking. NIPS.

[36] Polson NG, Scott JG, Windle J (2013). Bayesian inference for logistic models using pólya-gamma latent variables. J. Am. Stat. Assoc..

[37] Politis DN, Romano JP (1994). Large sample confidence regions based on subsamples under minimal assumptions. Ann. Stat..

[38] Politis DN, Romano JP, Wolf M (2001). On the asymptotic theory of subsampling. Stat. Sin..

[39] Geyer, C.J. 5601 notes: The subsampling bootstrap. *Unpublished manuscript* (2006).

[40] Fitzenberger B (1998). The moving blocks bootstrap and robust inference for linear least squares and quantile regressions. J. Econ..

[41] Meinshausen N, Bühlmann P (2010). Stability selection. J. R. Stat. Soc. Ser. B (Stat. Methodol.).

[42] Ahmed I, Pariente A, Tubert-Bitter P (2018). Class-imbalanced subsampling lasso algorithm for discovering adverse drug reactions. Stat. Methods Med. Res..

[43] Strathmann, H., Sejdinovic, D. & Girolami, M. Unbiased bayes for big data: Paths of partial posteriors. arXiv preprint arXiv:1501.03326 (2015).

[44] Knight K, Fu W (2000). Asymptotics for lasso-type estimators. Ann. Stat..

[45] Chatterjee A, Lahiri S (2010). Asymptotic properties of the residual bootstrap for lasso estimators. Proc. Am. Math. Soc..

[46] Chatterjee A, Lahiri SN (2011). Bootstrapping lasso estimators. J. Am. Stat. Assoc..

[47] Campbell M, Donner A, Klar N (2007). Developments in cluster randomized trials and statistics in medicine. Stat. Med..

[48] Carnegie NB, Wang R, De Gruttola V (2016). Estimation of the overall treatment effect in the presence of interference in cluster-randomized trials of infectious disease prevention. Epidemiol. Methods.

[49] Staples PC, Ogburn EL, Onnela J-P (2015). Incorporating contact network structure in cluster randomized trials. Sci. Rep..

[50] Wang R, Goyal R, Lei Q, Essex M, De Gruttola V (2014). Sample size considerations in the design of cluster randomized trials of combination hiv prevention. Clin. Trials.

[51] Green PJ (1995). Reversible jump markov chain monte carlo computation and bayesian model determination. Biometrika.

[52] Park T, Casella G (2008). The bayesian lasso. J. Am. Stat. Assoc..

[53] Casella G, George EI (1992). Explaining the gibbs sampler. Am. Stat..

[54] Green PJ, Hastie DI (2009). Reversible jump mcmc. Genetics.

[55] Gelman A, Rubin DB (1992). Inference from iterative simulation using multiple sequences. Stat. Sci..

[56] Brooks SP, Gelman A (1998). General methods for monitoring convergence of iterative simulations. J. Comput. Graph. Stat..

[57] Gelman A, Carlin JB, Stern HS, Rubin DB (2014). Bayesian data analysis.

[58] Castelloe, J.M. & Zimmerman, D.L. Convergence assessment for reversible jump mcmc samplers. *Department of Statistics and Actuarial Science, University of Iowa, Technical Report* **313** (2002).

[59] Brooks SP, Giudici P (2000). Markov chain monte carlo convergence assessment via two-way analysis of variance. J. Comput. Graph. Stat..

[60] Efron B, Hastie T, Johnstone I, Tibshirani R (2004). Least angle regression. Ann. Stat..

